# Factors associated with psychological and behavioral functioning in people with type 2 diabetes living in France

**DOI:** 10.1186/1477-7525-8-124

**Published:** 2010-11-02

**Authors:** Stephanie Boini, Marie-Line Erpelding, Anne Fagot-Campagna, Mounir Mesbah, Judith Chwalow, Alfred Penfornis, Vincent Coliche, Etienne Mollet, Keith Meadows, Serge Briançon

**Affiliations:** 1 Clinical Epidemiology and Evaluation, CIC-EC CIE6 Inserm, University hospital of Nancy, France; 2 Nancy University, P. Verlaine - Metz University, Paris - Descartes University, EA 4360 Apemac, Nancy, France; 3 Department of Chronic Disease and Injury, Institute for Health Surveillance (InVS), Saint-Maurice, France; 4 Theoretical and Applied Statistics Unit, Pierre & Marie Curie University - Paris VI, Paris, France; 5 National Federation of the Blind, Baltimore, USA; 6 Diabetology - Endocrinology - Nutrition - Metabolism, University Hospital of Besançon, France; 7 National Association for Diabetes Networks Coordination (ANCRED), Paris, France; 8 DHP Research & Consultancy Limited, Tower Hamlets PCT, London, UK

## Abstract

**Background:**

To identify demographic and clinical factors associated with psychological and behavioral functioning (PBF) in people with type 2 diabetes living in France.

**Methods:**

In March 2002, approximately 10,000 adults, who had been reimbursed for at least one hypoglycemic treatment or insulin dose during the last quarter of 2001, received a questionnaire about their health status and PBF (3,646 responders). For this analysis, the 3,090 persons with type 2 diabetes, aged 18-85 years old were selected.

PBF was measured with the adapted version of the Diabetes Health Profile for people with type 2 diabetes. This permitted the calculation of three functional scores - psychological distress (PD), barriers to activity (BA), and disinhibited eating (DE) - from 0 (worst) to 100 (best).

**Results:**

Major negative associations were observed with PBF for microvascular complications (a difference of 6.7 in the BA score between persons with and without microvascular complications) and severe hypoglycemia (difference of 7.9 in the BA score), insulin treatment (-8.5 & -9.5 in the PD & BA scores respectively, as compared to treatment with oral hypoglycemic agents), non-adherence to treatment (-12.3 in the DE score for persons forgetting their weekly treatment), increasing weight (-8.5 & -9.7 in the PD & DE scores respectively, as compared to stable weight), at least one psychiatrist visit in 2001 (-8.9 in the DE score), and universal medical insurance coverage (-7.9 in the PD score) (due to low income).

**Conclusion:**

Prevention and management of microvascular complications or adherence to treatment (modifiable factors) could be essential to preserving or improving PBF among people with type 2 diabetes. A specific approach to type 2 diabetes management may be required in groups with a low socioeconomic profile (particularly people with universal medical insurance coverage), or other non modifiable factors.

## Background

At least 33 million people in European countries had diabetes in 2000. This number will be multiplied by 1.5 and will reach 48 million by 2030. A similar trend is observed in France [1]. The prevalence of diabetes is estimated at 3.95% and the yearly increase of the prevalence is at 5.7%. Six percent of mortality is related to diabetes and 5% of the resources of the health care system are used by people with diabetes [2]. Diabetes is a "fraught with consequences" chronic disease due to its life-threatening complications and reduced life expectancy. It may develop from a non-symptomatic disease, during which patients must adhere to bothersome and difficult therapy, to a far advanced one, with serious micro- and macro-vascular complications. Diabetes negatively affects physical (development of short- and long-tem complications, symptoms) [3], psychological (depressed mood, fatigue, frustration, anxiety) [4,5] and social (change in the quantity and the quality of patients relationships) functioning [6]. Medical care, while not able to cure most chronic diseases, can limit their impact on a patient's daily life. Diabetes is a typical chronic disease that places a burden on the person's daily activities. Considering the patient's point of view is thus essential in the care and treatment of diabetes. To this end, both generic as well as diabetes specific health-related quality of life (HRQoL) instruments were developed. Studies focusing on correlates of HRQoL in people with diabetes have underlined the importance of complications [7-9] and depression [10-13].

In France, in 2001, however, there were few data concerning the frequency of diabetes specific complications, prevention and treatment of these complications, and even less concerning patient perceptions about diabetes as a condition and its treatment. For this reason, the Institute for Health Surveillance, the major national health insurance system in France and the National Association for Diabetes Networks Coordination initiated a large study named ENTRED [14], which included information about diabetes-related psychological and behavioral functioning (PBF).

The aims of this study were to measure psychological and behavioral functioning (PBF) levels of people with type 2 diabetes living in France, and to identify socio-demographic and clinical factors associated with PBF in this population.

## Methods

### Data source and subjects

The data analyzed here were drawn from the ENTRED study.

ENTRED was designed to characterize, to evaluate and to monitor the health status and the management of patients treated for diabetes and living in France.

Briefly, subjects were randomly selected from the national health insurance system database which covers about 70% of the French population: 10,000 adults living in France, who had been reimbursed for at least one hypoglycemic treatment or insulin dose during the last quarter of 2001, received a mailed questionnaire about their health status and PBF in March 2002. Thirty six point five percent (n = 3,646) of the questionnaires were returned. In addition, data from all medical reimbursements were used to characterize markers of medical care consumption during the year 2001.

The study was approved by the local Institutional Review Board (CCTIRS n° 01170) and the "Comité National Informatique et Liberté" (CNIL n°901236), which ensures the confidentiality of all information collected.

Data collection concerned both people with type 1 and type 2 diabetes. People with oral hypoglycemic treatment or those for whom diabetes had been diagnosed after 45 years of age or for whom diabetes had been diagnosed before 45 years of age with insulin treatment started at least 2 years after diagnosis, were considered as people with type 2 diabetes. Ninety one point two percent of respondents were classified as type 2 and 6.4% as type 1 (2.4% were unclassified due to missing information or presence of another type of diabetes).

Only people up to 85 years old with type 2 diabetes (n = 3,090) were taken into account in this analysis. People with type 1 diabetes (n = 231) were excluded due to major differences in age as well as PBF [15]. Factors associated with type 1 diabetes are reported elsewhere [16]. People over age 85 years (n = 66) were excluded due to the potential lack of validity of their answers. The sample characteristics are summarized in table 1.

**Table 1:** Characteristics of people with type 2 diabetes (n = 3,090)

	n (%)	
**Female**	1,405	(45.5%)
**Age **(mean ± sd)	63.9 ± 10.4	
**Living alone**	780	(25.6%)
**Education level**		
Less than high school	1,611	(60.6%)
High school	802	(30.2%)
University	245	(9.2%)
Missing	432	
**Current professional activity**		
Employed	514	(16.8%)
Retired	2,084	(68.0%)
Other	466	(15.2%)
**Country of birth**		
France	2,468	(79.9%)
North Africa	335	(10.8%)
Europe	143	(4.6%)
Other country	144	(4.7%)
		
**Diabetes duration **(mean ± sd)	11.2 ± 9.5	
0-4 years	868	(29.7%)
5-9 years	575	(19.7%)
10-19 years	866	(29.6%)
20 years and more	615	(21.0%)
**Diabetes treatment**		
Oral hypoglycemic agents	2,565	(83.0%)
Insulin	307	(9.9%)
Insulin & Oral hypoglycemic agents	218	(7.1%)
		
**DIABETES COMPLICATIONS**		
At least one severe hypoglycemic episode	300	(10.0%)
At least one macrovascular complication ^a^	589	(19.5%)
At least one microvascular complication ^b^	341	(11.8%)

ENTRED 2001

^a ^Presence of myocardial infarction, angina or heart arteries surgery

^b ^Presence of foot ulcer, amputation, eye vision loss, dialysis or kidney transplantation

### Data collection

#### Outcomes of interest: psychological and behavioral functioning

These were measured using the adapted version of the Diabetes Health Profiles DHP-1 questionnaire for people with type 2 diabetes, the DHP-18 [17]. The 18 items were summed into three subscale scores: psychological distress -PD (6 items); barriers to activity -BA (7 items); and disinhibited eating -DE (5 items).

Analyses were performed which support the validity of the French DHP-18 version: the same 3 dimensions of the original version were identified, internal consistency was estimated as good, items-to scale correlations between items and their own dimension were high and those between items and the 2 other dimensions were low, floor and ceiling effects were limited and acceptability was considered as correct (not shown).

All three dimensions were scored from 0 to 100 (worst to best possible functioning). Scores were calculated as the mean of item values when more than half of the items was answered, and scores were recorded as missing elsewhere.

#### Socio-demographic and clinical characteristics of the subjects

These data were collected through the mailed questionnaire filled in by participants. Socio-demographic variables included: gender, age, living alone (yes/no), country of birth (further grouped into France, North Africa, Europe, Other), educational level (less than high school, high school, university), current professional activity (employed/retired/other), universal medical insurance coverage(yes/no), which, in France, permits free access to medical care for people with a low socioeconomic level.

Diabetes and lifestyle characteristics included: duration of diabetes and its treatment, self-reported weight and height which permitted the calculation of body mass index (BMI), changes in weight ("Currently, do you estimate that your weight is?": decreasing/increasing/stable), smoking ("Do you usually smoke?"), alcohol consumption, self-reported high blood pressure ("Has a doctor ever told you that you had high blood pressure?") and high cholesterol ("Has a doctor ever told you that you had too much cholesterol?"), regular physical activity ("Do you regularly practice physical activity at home, at work or during your leisure time?"), at least one dietician visit in 2001 (yes/no), weight-loss diet (yes/no).

Health status data about diabetes complications were used to construct 3 indicators: at least one microvascular complication ("Have you had foot ulcer", "... amputation", "...eye vision loss?", "Are you in dialysis or have you had a kidney transplantation?"); at least one macrovascular complication ("Has a doctor told you that you have had a myocardial infarction, angina?", "Have you had a surgery intervention on your heart arteries?"); at least one severe hypoglycemic episode ("Have you had any severe hypoglycemia, requiring the help of another person to raise your blood sugar?") in 2001.

Other variables recorded concerned adherence to treatment with insulin or oral hypoglycemic agents (OHAs) ("Do you forget your insulin or tablet?": weekly/monthly/less than monthly/never), the number of psychiatrics visits, endocrinology visits or hospitalization (one day or more), and glycated hemoglobin (HbA1c) test (analyzed as a dichotomous variable: < 3 vs. ≥3/year, cut-off defined according to the recommendations of the national agency of accreditation and evaluation in health care in 2001).

#### Medical reimbursements records from the health insurance system database

The number of general practitioner visits during the year 2001 and consumption of psychotropic drugs during the last quarter of 2001 were extracted from this database.

### Statistical Analysis

All descriptive statistics were presented as means and standard deviations for continuous variables, and as absolute and relative frequencies for categorical variables. DHP-18 scores were presented as means and standard deviations. Percent of missing scores were also calculated.

The relationships between each DHP-18 score (outcome) and each collected factor (i.e. sociodemographics, lifestyle characteristics, diabetes characteristics and complications from the patient questionnaire; medical care counsumption from the health insurance system database) were first tested by using analysis of variance (in the case of categorical independent variables) or simple linear regression (in the case of continuous independent variables). Then for each DHP-18 score, all significant factors were introduced in a multi-factor analysis of variance model and were removed from the final model when they were no longer associated with the considered DHP-18 score (backward selection).

The statistical threshold for significance was set at 0.05.

These three final multi-factor models were systematically adjusted for gender and age. Moreover, interactions between all significant factors and age and gender were tested for each model.

Statistical analysis was performed using SAS^(r) ^9.1 system software.

## Results

The mean scores of the three DHP-18 dimensions were: 76.8 ± 18.0 for the BA dimension, 66.4 ± 23.9 for the DE dimension and 80.5 ± 18.8 for the PD dimension. Percents of missing scores were lower than 10% (6.2%, 6.6% and 2.5% for the BA, DE and PD dimensions, respectively). Percents of missing raw items of the PD dimension were always under 5%; those of the BA and DE dimensions were around 7%.

### Factors associated with psychological and behavioral functioning

All sociodemographic, lifestyle, medical and clinical factors collected were significantly related to the one or the other of the 3 dimensions, except the tobacco status (not shown). The results of the multi-factor analyses are presented in table 2.

**Table 2:** Factors associated with the three DHP-18 scales, in people with type 2 diabetes

	Barriers to activity (N = 2,081)			Psychological distress (N = 2,113)			Disinhibited eating (N = 2,312)		
	
	beta	Se	p	beta	se	p	beta	se	p
**Intercept**	94.99	1.38	**< .001**	96.64	1.54	**< .001**	82.27	1.38	**< .001**
**Female**	1.29	0.76	**0.09**	- 4.86	1.80	**0.007**	- 5.25	1.01	**< .001**
**Age (1 yr)**	- 0.04	0.05	**0.44**	0.27	0.04	**< .001**	0.32	0.05	**< .001**
**Educational level**			**< .001**			**< .001**			
Less than high school	- 5.48	1.14	< .001	- 4.80	1.23	< .001			
High school	- 2.54	1.17	0.03	- 2.26	1.27	0.08			
University	0.00			0.00					
**Country of birth**						**0.02**			
Europe				- 3.41	1.75	0.05			
France				0.00					
North Africa				- 3.43	1.34	0.01			
Other				- 0.60	1.72	0.73			
**Living alone**				3.77	0.84	**< .001**	- 2.61	1.10	**0.02**
**Universal medical insurance coverage**	- 5.59	1.76	**0.002**	- 7.89	1.93	**< .001**			
**Diabetes duration (years)**			**0.04**			**< .001**			
0-4	0.00			0.00					
5-9	- 1.69	0.94	0.07	- 0.98	1.22	0.42			
10-19	- 2.32	0.87	0.008	- 2.16	1.10	0.05			
20 and more	- 2.37	1.03	0.02	- 2.20	1.28	0.09			
**Diabetes treatment**			**< .001**			**< .001**			**0.10**
Insulin	- 9.49	1.23	< .001	- 8.54	1.94	< .001	- 3.35	1.61	0.04
Insulin & OHAs	- 8.79	1.32	< .001	- 2.43	1.97	0.22	- 1.64	1.89	0.39
OHAs	0.00			0.00			0.00		
**Macrovascular complication ^a^**	- 3.00	0.90	**< .001**						
**Microvascular complication ^b^**	- 6.65	1.13	**< .001**	- 3.33	1.20	**0.006**			
**Severe hypoglycemia in 2001**	- 7.90	1.19	**< .001**						
**Weight-loss diet**	- 4.10	0.76	**< .001**				- 6.35	1.03	**< .001**
**At least one dietician visit in 2001**	- 2.27	0.82	**0.006**	- 4.31	0.85	**< .001**			
**Perceived weight variation**						**< .001**			**< .001**
Decreasing				- 0.81	1.90	0.67	- 1.45	1.39	0.30
Increasing				- 8.46	1.96	< .001	- 9.65	1.42	< .001
Stable				0.00			0.00		
**Insulin or Tablet forgetting**									**< .001**
Weekly							- 12.34	2.39	< .001
Monthly							- 5.96	1.76	< .001
Less than monthly							- 4.62	1.41	0.001
Never							0.00		
**Body Mass Index (1 kg/m²)**							- 0.51	0.16	**0.001**
**No regular physical activity ^c^**							- 6.25	1.78	**< .001**
**Alcohol consumption**			**< .001**			**0.03**			
Daily or almost daily	0.00		.	0.00					
Weekly	- 0.20	0.92	0.83	- 1.79	1.11	0.11			
Monthly	- 2.14	1.14	0.06	- 3.95	1.57	0.01			
Less than monthly	- 1.18	1.16	0.31	- 1.56	1.79	0.39			
Never	- 5.86	1.04	< .001	- 5.46	1.66	0.001			
**High level of cholesterol**	- 1.77	0.66	**0.008**	- 2.00	0.71	**0.005**	- 3.05	0.91	**< .001**
**At least one psychiatrist visit in 2001**	- 4.56	1.52	**0.003**				- 8.71	2.16	**< .001**
**Psychotropic substances delivery during the last quarter 2001**				- 2.82		0.79		**< .001**	
**Other(s) disease(s)**	- 3.64	0.68	**< .001**	- 4.06	0.79	**< .001**	- 4.38	0.92	**< .001**
**R² (%)**	**25**			**21**			**18**		

ENTRED 2001

Empty cells mean that factors were not included in the final multivariable model.

Underlining indicates the most important association with the DHP scores.

^a ^Presence of myocardial infarction, angina or heart arteries surgery; ^b ^Presence of foot ulcer, amputation, eye vision loss, dialysis or kidney transplantation; ^c ^House working, do-it-yourself, walking, building/unskilled working, or sport.

Significant interactions were found in the three dimensions:

- barriers to activity: age*other(s) disease(s), p < .0001; age*hypoglycemia, p = 0.006; age*treatment, p < .001;

- psychological distress: Sex*Alcohol consumption, p = 0.05; Age*Hypoglycemic episode, p = 0.01; Age*Treatment, p = 0.008; Diabetes duration*Hypoglycemic episode, p = 0.002; Diabetes duration*Psychiatrist visit, p = 0.04; Diabetes duration*Weight, p = 0.01; Treatment*Other(s) disease(s), p = 0.008;

- disinhibited eating: Sex*No regular physical activity, p = 0.005; Age*Living alone, p = 0.03; BMI*Diabetes duration, p = 0.004; BMI*Treatment, p = 0.005.

Thirteen factors were statistically associated with the BA dimension. In particular, patients treated with OHAs alone presented consistently higher scores on the BA dimension across all age groups, than patients on insulin or a combination of insulin and OHAs. This difference was more pronounced with older patients. Other important factors were a severe hypoglycemic episode during the previous year (-7.9 in the mean score as compared to subjects without severe hypoglycemic episodes; with a more pronounced negative association in younger people) and existing microvascular complication (-6.7), as well as universal medical insurance coverage (-5.6), a lower educational level (-5.5) and no alcohol consumption (-5.9). All these factors explained 25% of the variability.

In the PD dimension, 15 factors were related to psychological functioning. The maximal negative association with PD dimension was observed for insulin treatment (-8.5 as compared to treatment with OHAs), increasing weight (-8.5 as compared to stable weight) and universal medical insurance coverage (-7.9). The effect of the other factors was limited to a maximum of 5 point variations. The negative association of a severe hypoglycemic episode with PD was more pronounced in younger people. Twenty-one percent of the variability was explained by these factors.

In the DE dimension, 11 factors were associated with behavioral functioning. Higher associations were observed for non-adherent patients (-12.3 for patients who forgot their weekly treatment vs. never), people with an increasing weight (-9.7 as compared to stable weight), on a weight-loss diet (-6.3), sedentary people (-6.3), higher BMI, people who consulted a psychiatrist at least once in 2001 (-8.7). Overall, 18% of the variability was explained by all these factors.

## Discussion

Psychological and behavioral functioning was measured in a large sample of people with type 2 diabetes who were living in France by using a scale specific to diabetes. The original DHP-18 is a validated instrument for use with people with type 2 diabetes [17]. Concerning the French version, in line with Meadows' preliminary results (based on the responses of people with type 1 & 2 diabetes) [18], analyses performed only on people with type 2 diabetes support its validity. Scores observed in our sample were close to scores observed in people treated by insulin, OHAs or diet in previous studies [17,19]. As in our study, insulin therapy was associated with a marked decrease in psychological functioning.

Many factors were associated with the three DHP-18 dimensions in this sample: some were specific to the illness, some were related to socio-demographic factors, and some to health-related behavioral factors. The effect size, a distribution-based indicator, was calculated to determine whether a difference could be considered as important [20]. Accordingly, major negative associations (at least a difference of 5 between groups in the DHP score, corresponding here to small to medium effect size) were observed for major microvascular complications (effect size of 0.18) -with little or no effect for macrovascular complications- and for severe hypoglycemia (effect size of 0.24), insulin treatment (effect size from 0.20 to 0.32 according to the DHP score), non-adherence to the treatment (effect size of 0.45), increasing weight (effect size of 0.18), at least one psychiatrist visit (effect size of 0.25), and surprisingly no alcohol consumption (effect size from 0.19 to 0.25 according to the DHP score). Finally, universal medical insurance coverage (effect size from 0.20 to 0.46 according to the DHP score), which, in France, permits free access to medical care for people with a low socioeconomic level, was negatively associated with psychological functioning, suggesting a higher toll of diabetes in people with low socioeconomic level. According to Cohen, effect sizes of 0.2, 0.5 and 0.8 are considered as small, medium and large, respectively [20]. Therefore, in summary, the effect on PBF of non-adherence to treatment and universal medical insurance coverage can be considered as medium. The effect of insulin treatment, severe hypoglycemia, at least one psychiatrist visit and no alcohol consumption can be considered as small.

Our results are in line with those of others studies, that found a negative impact on HRQoL (especially on psychological functioning) of the presence of complications, comorbidities, depressive symptoms, insulin use, high BMI or a lower educational level [7-9,19,21]. Maddingan et al found that comorbidities (in particular depression and stroke) and markers of socioeconomic status were important factors related to HRQoL as measured by the health utilities index mark 3. To some extent, a lower educational level, longer diabetes duration, insulin use, higher BMI and non-practice of physical activity were also negatively associated with HRQoL in this study [22]. Others underlined the negative role of micro- or/and macro-vascular complications on HRQoL [6]. Surprisingly, compared to the impact of microvascular complications, the effect of macrovascular complications was limited (about a 3-decrease only in the BA dimension). Macrovascular disease was defined in this study as myocardial infarction or angina, or heart arteries surgery, and no distinction could be performed between myocardial infarction and angina. Moreover, no information about the time of occurrence was available. The presence of both complications was associated with a more important decrease than a single type of complication.

Diabetes complications contribute to excess morbidity and mortality and generate substantial costs. In order to provide timely treatment, it is essential that patients at risk for the development of diabetes complications are identified as early as possible. The normalization of factors such as blood pressure, blood cholesterol and plasma glucose can prevent or delay diabetes complications [3].

We found a major negative association between the DE score and having had at least one visit to the psychiatrist in 2001, which might reflect mental health problems. In particular, depression in diabetes can be approached in terms of depressed mood and anhedonia, cognitive symptoms and anxiety [5]. Depression is a well-recognized determinant of HRQoL in diabetes [10-12]. Identification and thus optimal care of depressive symptoms is important as depression is associated with poor diabetes self-management, an increased risk for complications, a lowed use of health services, increased functional impairment and distress may well impact the course of the illness [11,23,24].

Surprisingly, compared to no consumption, daily alcohol consumption was associated with better psychological functioning (BA and PD dimensions). There is some evidence that moderate alcohol consumption is associated with a lower risk of mortality and coronary heart disease in people with type 2 diabetes [25]; other studies suggested a beneficial effect of moderate alcohol consumption on glycemic control but this has not yet been demonstrated. Moderate alcohol consumption may also be a marker of specific psychological profiles.

The most important deficits observed in our study suggest that prevention and management of modifiable factors (for example microvascular complications or adherence to treatment) could be essential to preserving or improving psychological and behavioral functioning among people with type 2 diabetes. A specific approach to type 2 diabetes management may also be required in groups with a low socioeconomic profile (i.e. people with a low educational level or for those who are on universal medical insurance coverage) or other non modifiable factors. Data issue from ENTRED showed that people with a lower socioeconomic status have more frequent macrovascular complications and a lower quality of diabetes care [26]. Efforts to improve the prevention of complications, therapeutic education and diabetes management are required in this vulnerable population.

Diabetes management is complex and should be empowered early. To achieve this goal, a multidisciplinary approach is required. Medical interventions are needed in order to address a broader spectrum of outcomes such as patient-reported outcomes (e.g. HRQoL), personal models of illness and empowerment. The promotion of self-management using strategies that take into account the adaptation to the illness and its treatment (stress management for self-care of the disease, psycho-behavioral methods, psychosocial support...) that are not only limited to drug therapy, should be encouraged [27]. Four groups of factors accounted for most of the variability in self-care behavior in patients with diabetes: patient characteristics, the patient family, the practitioner and the health system, and the community/work setting [28]. Integrating such factors in the disease management can only be beneficial for patients.

There are some limitations in this study. The cross-sectional design did not allow us to examine any causal effect. Data were self-reported and people could under or over-report any of the collected conditions. However, when analyzing the same data collected through a medical questionnaire (sent to the responders' physician when its address was reported on the participant's questionnaire), Romon *et al *compared the prevalence of the macrovascular complications based on both the patients and physicians declarations [29]. Estimates were similar overall, whether they were reported by patients or physicians. Moreover, Martin *et al *found that self-reports are reasonably accurate for certain chronic conditions and for routine screening exams [30]. The algorithm used to distinguish between responders with type 1 and type 2 diabetes may have contributed to some misclassification of the different types of diabetes. This could affect the validity of our results if the factors associated with PBF differed between the two diseases.

External validity of our results is another important question. People treated with diet only could not be included because the sample selection was based on the database of the national health insurance system, which covered only reimbursed medical prescriptions. They may represent about 10% of people with type 2 diabetes [31] and have been reported to show similar HRQoL as people treated with OHAs [6]. Our sample showed similar characteristics with other French samples in terms of age, sex, educational level, BMI, smoking [31-33]. The response rate was about 40%, a level generally observed in this type of mailing survey. The characteristics of the responders and the non-responders were compared using medical claims, available for all [29]. In our sample, responders were younger and more often male, more frequently treated with insulin or with several OHAs than with a single OHAs, less frequently treated for a cardiovascular disease and received an overall better quality of diabetes care [29]. Our results may therefore underestimate the true impact of diabetes on PBF.

## Conclusion

Factors associating with PBF were numerous, variable and sometimes specific to one or the other of the three dimensions. Particularly, prevention of diabetes microvascular complications and severe hypoglycemic episodes, as well as improvement of patient adherence, with a special attention to vulnerable (i.e. with a low socioeconomic profile) people with type 2 diabetes, should be sought.

In 2006, the French state launched a specific plan to improve HRQoL in people with chronic disease. A better understanding of the broader factors associated with PBF is the first step necessary to permitting the development of interventions and policies that will preserve or improve the daily lives of people with type 2 diabetes. Attention to these markers could lead to an improvement in functioning. In 2007, ENTRED will be repeated in order to measure the possible evolution since 2001. Moreover, the use of the SF12, a generic instrument will permit a comparison of HRQoL within the general French population.

## Abbreviations

HRQoL: health-related quality of life; PBF: psychological and behavioral functioning; DHP: diabetes health profile; SF12: short form 12 items questionnaire; PD: psychological distress; BA: barriers to activity; DE: disinhibited eating; OHA: oral hypoglycemic agents; HbA1c: glycated hemoglobin.

## Competing interests

The authors declare that they have no competing interests.

## Authors' contributions

SBo participated in the design of this ancillary work, reviewed the literature and drafted the manuscript. MLE participated in the design of this work, performed the statistical analysis, and provided feedback on this work. MM, JC, AP, VC, EM, and KM provided feedback on this work. JC contributed to and revised the English version. AFC and SBr participated in the design of this work and provided feedback on it. All authors collaborated interactively, read and approved the final version.
